# Exploratory pilot study of minimally invasive therapy: laser ablation combined with acellular dermal matrix implantation for recurrent sacrococcygeal pilonidal disease

**DOI:** 10.3389/fbioe.2026.1792099

**Published:** 2026-05-07

**Authors:** Zhicheng Li, Caizhong Lu, Lei Jin, Xingli Xu, Jialin Qin, Zhenyi Wang, Jiong Wu

**Affiliations:** 1 Department of Proctology, Yueyang Hospital of Integrated Traditional Chinese and Western Medicine, Shanghai University of Traditional Chinese Medicine, Shanghai, China; 2 Department of Proctology, Shanghai Pudong New Area Guangming Hospital of Traditional Chinese Medicine, Shanghai, China

**Keywords:** acellular dermal matrix, laser ablation, minimally invasive surgery, preliminary evidence, recurrent sacrococcygeal pilonidal disease

## Abstract

**Purpose:**

Recurrent sacrococcygeal pilonidal disease (SPD) poses significant therapeutic challenges. Recently, minimally invasive techniques, notably laser ablation, have emerged as promising alternatives to conventional surgery. Concurrently, the application of acellular dermal matrix (ADM) has shown potential in facilitating wound healing and tissue regeneration. This small retrospective exploratory pilot study aims to evaluate the efficacy and safety of a combined therapeutic approach utilizing laser ablation and ADM implantation for the management of recurrent SPD.

**Methods:**

We retrospectively analyzed clinical data of our five patients who diagnosed with recurrent SPD between October 2023 and April 2024. All patients underwent laser ablation combined with ADM placement. Clinical outcomes, including healing rates, recurrence rates, and operative data, were recorded. Postoperative pain was quantified using the Visual Analog Scale (VAS), and the time to return to work and daily activities was documented. No sample size calculation was performed due to the exploratory nature of the study.

**Results:**

A total of five patients underwent laser ablation combined with ADM. The median time to return to work was 10 (9, 11) days, and complete wound healing was achieved in a median of 34 (30, 35) days. Patients experienced minimal postoperative pain. Over a median follow-up period of 7 (6–7) months, all patients were successfully cured without recurrence and satisfied with this surgery.

**Conclusion:**

Preliminary evidence suggests that laser ablation combined with ADM constitutes a feasible and promising minimally invasive strategy for recurrent SPD. This technique is associated with rapid wound healing, negligible pain, and the absence of recurrence in this cohort. This study is a small-sample retrospective pilot research with inherent limitations, and the findings warrant further verification by large-sample studies with medium and long-term follow-up.

## Introduction

SPD is an infectious condition affecting the gluteal cleft, presenting acutely with swelling and pain. While the acute phase causes significant morbidity, the disease frequently progresses to a chronic sinus tract refractory to healing. This condition predominantly affects adolescents and young adults, with an increasing incidence rate. The standard of care for symptomatic disease typically involves surgical excision, with or without primary closure. However, such interventions often result in substantial tissue defects, prolonged convalescence, and demanding postoperative nursing care (Al-Khamis et al., 2010).

Although various minimally invasive surgical techniques have been introduced, recurrence remains a significant concern, with rates reaching up to 68% over 10 years depending on the modality used (Stauffer et al., 2018). Currently, there is no optimal consensus on the management of recurrent SPD. Repeated excision or suturing may adversely impact quality of life. Consequently, there is an urgent need for effective, minimally invasive surgical strategies.

Since 2019, our team has utilized 1470-nm radial diode laser fibers for SPD treatment, successfully treating over 500 patients, except those in the acute abscess stage. Clinical practice has confirmed that laser ablation is an effective minimally invasive technique, characterized by mild postoperative pain, minimal tissue trauma and high patient satisfaction (Li et al., 2023; Dessily et al., 2019). The laser emits radial energy that uniformly targets the epithelial lining of the sinus tract, inducing thermal destruction and subsequent tract obliteration. However, some patients are prone to infection and recurrence, attributed to factors such as the large cavity within the sinus tract, the slow regeneration of new tissue, and incomplete tissue filling within the tract postoperatively.

ADM, a biological scaffold derived from the dermis, supports host cell ingrowth, wound repair, and capillary regeneration. Additionally, ADM possesses anti-infective properties and is widely employed in treating skin defects from burns, trauma, and soft tissue reconstruction (Gierek et al., 2022; Kirsner et al., 2015; Shitrit et al., 2014). Multiple studies have suggested that ADM can be used to treat complex anal fistulas without damaging the anal sphincter or anal shape, with cure rates ranging from 12.5% to 88% (Ellis et al., 2010; Schwandner et al., 2018; ba-bai-ke-re MM et al., 2010; Balcis et al., 2017). A small-sample study reported that the application of ADM in excisional surgery for recurrent SPD accelerated tissue proliferation, reduced wound healing time and prevented disease recurrence (Vaughn and Lalikos, 2011). We hypothesized that combining laser ablation with ADM could further accelerate healing, minimize pain, and reduce recurrence rates compared to laser ablation alone. This small-sample retrospective exploratory pilot study reports the short-term clinical outcomes of 5 patients with recurrent SPD treated with this novel combined approach.

## Materials and methods

This observational study included five patients diagnosed with recurrent SPD who had previously undergone surgical intervention (excluding simple abscess drainage). Inclusion criteria: (1) Aged 18–60 years; (2) Clinically and radiologically diagnosed with recurrent SPD (Huurman et al., 2024); (3) No acute abscess formation at the time of admission; (4) Voluntarily participated in the study and signed informed consent. Exclusion criteria: (1) Decompensated diabetes mellitus, immune insufficiency; (2) Complicated with other severe systemic diseases (e.g., severe cardiovascular and cerebrovascular diseases, liver and kidney failure); (3) Allergic to laser treatment or ADM materials; (4) Pregnant or lactating women. Specifically, two patients had previously undergone flap techniques, while three patients had undergone excision with primary closure. Informed consent was obtained from all participants. The surgeries were performed by the same physician.

## Surgical technique

**FIGURE 1:**
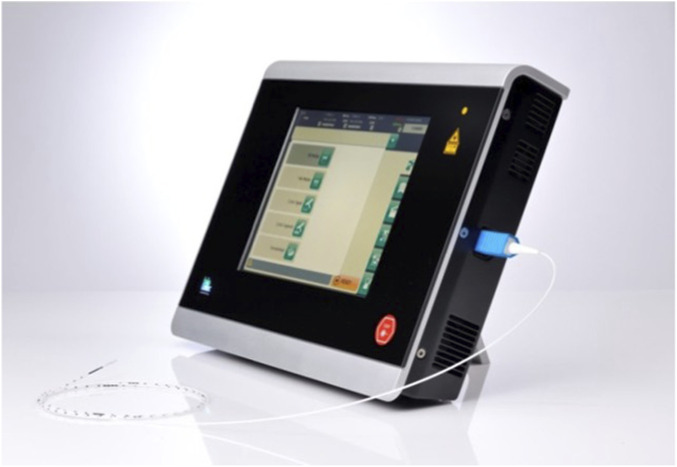
Biolitec device used for sinus laser treatment.

**FIGURE 2:**
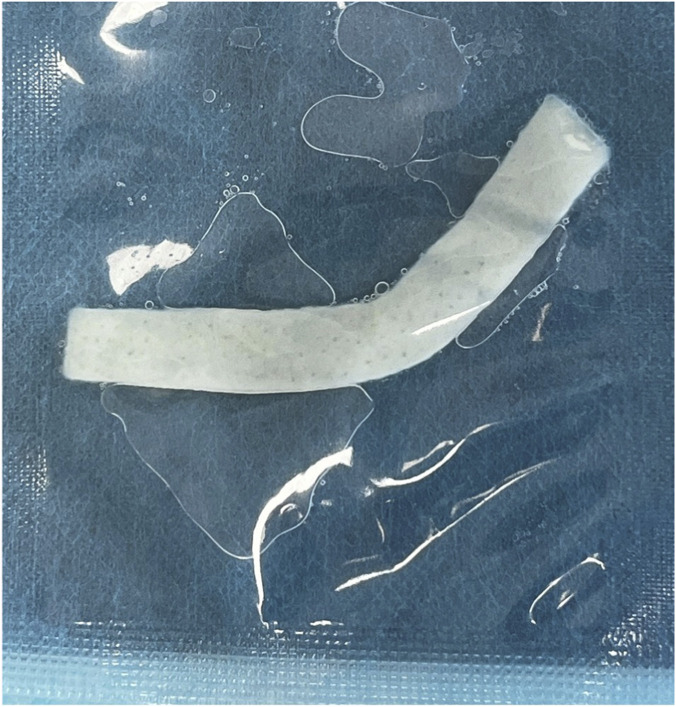
The ADM in packaging.

All patients underwent preoperative preparation of the sacrococcygeal area. Patients were placed in the left lateral position and intravenous anesthesia was induced and maintained using Propofol Medium/Long Chain Fat Emulsion Injection (Propofol was infused via Target-Controlled Infusion (TCI) after setting the patient’s age, height, and weight parameters, inducing at 3 μg/mL and maintaining at 2 μg/mL). A circular or elliptical incision was made at the top of the sinus tract, based on the extent of infection. A mosquito clamp was inserted through the incision to clear hair within the sinus tract. A probe was then inserted reversely from the incision to ascertain the course of the sinus tract, and an incision was made at the protruding end of the probe. All primary pits and secondary openings of the sinus tract were systematically identified and incised to ensure complete exposure. No additional auxiliary maneuvers such as curettage, irrigation or methylene blue dyeing were used in this study. A fiber-optic catheter (Figure 1) (from the German Biolitec Leonardo DUAL 45 laser device, Biolitec AG, Germany, featuring a single-loop laser catheter) was inserted into the sinus tract and guided by a light indicator to reach the end of the tract. Laser energy (10 W, 1,470 nm) was delivered to ablate the tract while withdrawing the fiber at a speed of approximately 1 mm/s, ensuring complete closure. Subsequently, a 10 cm × 10 cm ADM graft (Figure 2) (The ADM was provided by Beijing Jayyalife Biological Technology Co., Ltd (NMPA Certificate: 20,223,130,785), derived from pigs) was rehydrated in sterile saline for 5 min, and then trimmed to fit the size and shape of the surgical cavity. The graft was secured to the wound edges using interrupted 2–0 absorbable sutures which degrade naturally within 60–90 days and do not require removal.

**FIGURE 3:**
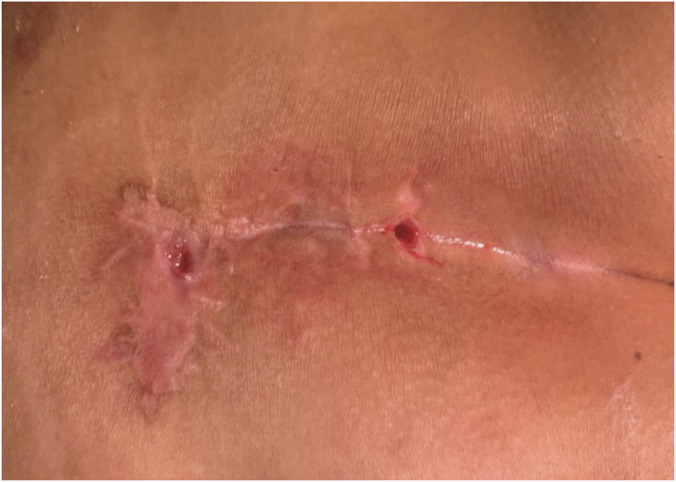
Preoperative picture.

**FIGURE 4:**
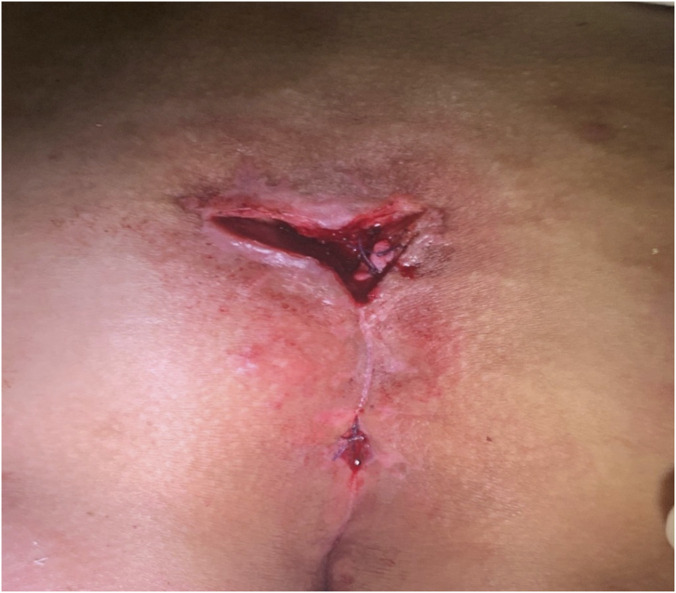
After the operation.

**FIGURE 5:**
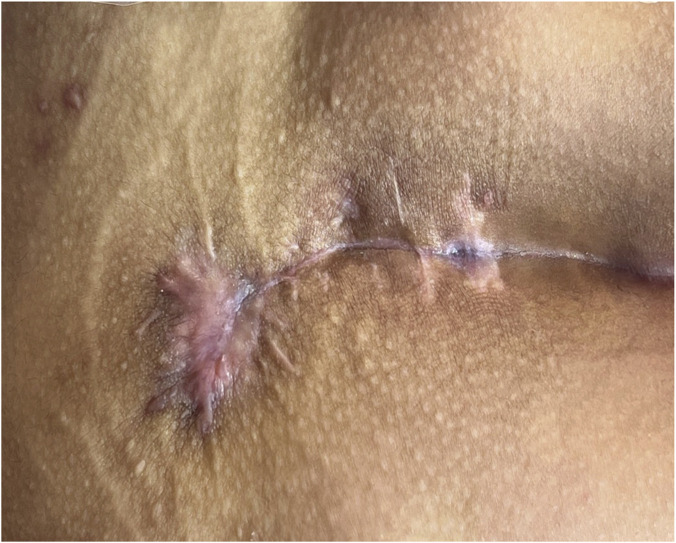
Successful healing at 30 days.

Postoperative care included disinfection of the wound with povidone-iodine, followed by applying a sterile gauze soaked with a small amount of Kangfuxin Liquid for wet compress on the wound, twice daily. Postoperatively, patients were instructed to keep the wound clean, avoid prolonged sitting and strenuous exercise until complete wound healing, and attend weekly follow-up via an online platform—where they uploaded wound photographs and described changes in their condition until healing was achieved. Images showing the condition before and after surgery are provided (Figures 3–5).

## Outcome measures

1. Operative Parameters: Laser ablation energy (Joules) and duration.
2. Postoperative Pain: Assessed using the Visual Analog Scale (VAS) by the attending surgeon on the day of surgery and postoperative days (POD) 1, 3, and 7 during dressing changes.
3. Complications: Incidence of wound infection or hemorrhage within the first postoperative week.
4. Recovery: Time to return to work and normal daily activities.
5. Healing: Defined as complete epithelialization with no discharge, absence of symptoms, and formation of a stable scar.
6. Recurrence: Suppurative manifestations such as ulceration and pus flow occur again at the same site after the wound surface is completely healed.

## Follow-up protocol

All patients were followed up according to a standardized schedule. Outpatient follow-up visits were scheduled at 1 week, 1 month, 3 months, and 6 months after surgery. During each follow-up, wound appearance, secretion, epithelialization, and scar status were evaluated and recorded. Any symptoms suggesting recurrence, such as swelling, pain, or discharge, were inquired and documented. For patients who could not attend in-person visits, clear digital photographs of the surgical area were obtained and reviewed by the attending surgeon to ensure assessment reliability. To minimize underestimation of asymptomatic recurrence, all patients were advised to seek immediate evaluation if any abnormal symptoms occurred between scheduled follow-up visits.

## Data analysis

Data analysis was conducted using SPSS 26.0 statistical software. Quantitative data that conform to a skewed distribution are expressed as M (Q1, Q3). Categorical data are presented as (n).

## Results

Five patients (4 males, 1 female) with a median age of 29 (25, 32) years underwent the combined procedure. The median operative time was 23 (19, 26) minutes, and the median sinus tract length was 6 (5, 8) cm. Total laser energy delivered was 374.7 (269.2–594.8) J. The median VAS score on the day of operation was 1 (1, 2), on the first postoperative day was 1 (1, 3), on the third postoperative day was 1 (1, 2), on the seventh postoperative day was 1 (1, 2). No postoperative complications occurred. The median time to return to work was 10 (9, 11) days, and complete wound healing was achieved in 34 (30, 35) days. Over a median follow-up period of 7 (6, 7) months, all patients achieved complete resolution with no recurrence. Patient satisfaction was 100% (Very Satisfied) (Table 1, 2).

**TABLE 1:** Baseline patients’ characteristics and postoperative outcome.

Characteristic	Value
Gender Male (%) Female (%)	5 4(83%) 1(17%)
Age (years) median (Q1, Q3)	29(25,32)
BMI (kg/m2) median (Q1, Q3)	23(22,27)
Sinus tract length (cm), median (Q1, Q3)	6(5,8)
Current smoker (%)	33.3%
Number of pits median (Q1, Q3)	1(0,1)
Number of previous operations median (Q1, Q3)	1(0,2)
Family history of pilonidal sinus disease (%)	0
Natal cleft hairiness (%) None/mild Intermediate Significant hairiness	1(33.3%) 4(6.7%) 0
Operative time (min) median (Q1, Q3)	23(19,26)
Energy (J), median (Q1, Q3)	374.7(269.2- 594.8)
VAS score, median (Q1, Q3) Operation day Postoperative (1 Day) Postoperative (3 Day) Postoperative (7 Day)	1(1,2) 1(1,3) 1(1,2) 0(0,0)
Patient satisfaction index n (%) Very satisfied Satisfied Neutral Dissatisfied Very dissatisfied	5(100) 0 0 0 0
Follow up(days) median (Q1, Q3)	7(6,7)
Complications, n (%)	0
Time(days)median (Q1, Q3) To complete healing To return to work	34(30,35) 10(9,11)
Recurrence (n)	0
Follow up (month) median (Q1, Q3)	14.5(13, 16)

**TABLE 2:** Baseline demographics and result.

Patient number	Age (years)	Sex	BMI	Duration of illness (months)	Results	Time to healing (days)
1	25	F	30.47	48	Healed	30
2	21	M	27.76	12	Healed	34
3	32	M	22.40	108	Healed	31
4	29	M	21.60	12	Healed	34
5	32	M	23.51	5	Healed	35

## Discussion

Despite the multitude of surgical methods for SPD, long-term recurrence rates remain problematic (Stauffer et al., 2018). Recurrent SPD presents a therapeutic dilemma, as no standardized guidelines currently exist. Chronic disease and repeated failures significantly impact patient psychology and self-esteem. An ideal method should minimize invasiveness while maximizing cure rates and patient satisfaction. Thus, minimally invasive surgeries are being increasingly used for pilonidal sinus disease (Agnesi et al., 2025).

Dr. Megha Shalini performed the Limberg procedure on 14 patients with recurrent SPD, with no recurrences observed postoperatively, and all patients returned to work within 3 weeks (Shalini et al., 2022). Postoperative pain was reported as mild, but this approach causes significant appearance damage and requires surgeon experience. Pronk treated 57 patients with recurrent SPD using phenolization, 51 patients resumed their normal daily activities 2 weeks later, with 90% of patients achieving complete wound healing after 3 months (Pronk et al., 2020). Despite its minimally invasive nature, phenolization is associated with a relatively low cure rate. The toxicity of phenol has led to its prohibition in certain countries (Iesalnieks et al., 2016).

Laser ablation, first proposed by Dessily et al., demonstrated an 87.5% success rate with a low recurrence rate of 2.9% (Dessily et al., 2017). This technique operates on the principle of uniformly applying circular energy emitted by a laser catheter to the epithelial cells lining the sinus tract, resulting in cellular damage and subsequent natural contraction of the sinus. Increasing numbers of clinical studies have confirmed the advantages of laser treatment (Sluckin et al., 2022; Algazar et al., 2022; Draullette et al., 2024; Horesh et al., 2023). However, longer-term follow-up has indicated recurrence rates rising to 15% within 6 months (Dessily et al., 2019). Our institutional experience mirrors these findings: while laser ablation is excellent for primary disease, recurrence in complex cases often stems from inadequate closure of large cavities. Conversely, as the number of patients increased and follow-up durations extended, the recurrence rate also demonstrated a relative upward trend. The rationale behind this is that, while laser treatment can damage the sinus wall tissue, it exhibits poor closure efficacy for sinus tracts with larger cavities. Inadequate postoperative care may result in delayed healing or recurrence. Nevertheless, laser surgery remains a viable option for recurrent patients without affecting the success rate of the surgery (Georgiou, 2018).

ADM is an innovative biocompatible material derived from allogeneic or xenogeneic skin tissue, which facilitates the repair and reconstruction of defective tissues. When utilized as a filler in sinus tracts, ADM can promptly induce connective tissue formation and neovascularization upon integration with the wound. The basement membrane side provides a natural substrate for the migration and colonization of epithelial cells, promoting epithelialization of the decellularized dermis, while the dermal side favors the ingrowth of host cells and rapid vascularization. Following transplantation, ADM can guide the infiltration of recipient cells and neovascularization, forming a new extracellular matrix (Israeli, 2012; Shridharani and Tufaro, 2012). It endows the tissue with toughness, elasticity, hydration, and shock absorption against mechanical forces, providing a microenvironment for cell survival and various activities (Munster et al., 2001). Eventually, it will degrade and be replaced by newly formed tissue. Its excellent biocompatibility and biodegradability render it effective in repairing dermal defect wounds, minimizing scar hyperplasia post-healing, and exhibiting certain anti-infective properties. It has been widely applied in various clinical departments. For complex anal fistula surgery with significant anal wound defects, ADM has also demonstrated effective reparative capabilities, they suggest that ADM is a reasonable new option for closure of anal fistulas (Gómez-Jurado et al., 2022; Han et al., 2009; Shelton and Welton, 2006). ADM has shown promise in reducing postoperative complications in complex hard-to-heal wounds and large cavity with inflammation through its ability to modulate the inflammatory response in these chronically inflamed soft tissues, fill surgical dead space and rapidly regenerate soft tissues. However, the clinical application of ADM may be limited by its relatively high cost and restricted availability in some regions. In China, the use of ADM does not impose a substantial medical economic burden on patients, which supports its clinical popularization.

Several studies have investigated the use of ADM as an auxiliary filler in resection and surgical methods for patients with recurrent SPD (Vaughn and Lalikos, 2011; Chaffin et al., 2021). Notably, these studies reported no postoperative recurrences, showed in Table 3. However, the patient’s postoperative experience was not as favorable as that observed with laser ablation alone. Our preliminary research indicates that a surgical approach combining laser ablation with ADM achieved a 100% cure rate, with minimal pain and no recurrences during the follow-up period, significantly reducing the time required for patients to resume their normal lives. Consequently, for complex sinus tract diseases with hard-to-heal wounds following repeated surgeries, the combination of laser ablation with ADM may not only enhance the patient experience but also potentially reduce the recurrence rate.

**TABLE 3:** Clinical Characteristics of SPD Patients Treated with ADM Implantation in Published Studies (NPWT: Negative pressure wound therapy; ECM: extracellular matrix).

Case (ID)	Gender/Age (y)	Past surgical history	Surgical method	Area of Diseased Tissue	Recurrence
Patient 1	M,21	Excision and primary closure, and excision And NPWT	Excision with placement of integra And NPWT	No mention	No
Patient 2	F,19	Primary closure, healing by secondary intention, and NPWT	Excision with placement of integra And NPWT	No mention	No
Patient 3	F, 21	Incision and drainage	Flap reconstruction with ECM + NPWT	7 × 11 cm	No
Patient 4	M, 20	Incision and drainage, excision and primary closure	Flap reconstruction with ECM + NPWT	12 × 6 cm	No
Patient 5	M, 19	Incision and drainage, excision and primary closure	Flap reconstruction with ECM + NPWT	12 × 3 cm	No
Patient 6	F, 52	Incision and drainage, excision and primary closure	Flap reconstruction with ECM + NPWT	10 × 4 cm	No
Patient 7	M, 19	Incision and drainage, excision and primary closure	Flap reconstruction with ECM + NPWT	11 × 4 cm	No
Patient 8	M, 15	Incision and drainage, excision and primary closure	Flap reconstruction with ECM + NPWT	12 × 5 cm	No

## Limitations of the study

This study has several inherent limitations that must be acknowledged, which severely restrict the generalization of the findings: (1) Extremely small sample size (n = 5) and retrospective study design, with no sample size calculation performed, leading to insufficient statistical power for robust conclusions on disease recurrence; (2) Short follow-up period: late recurrence is common in SPD, and the absence of recurrence in this short follow-up period does not guarantee long-term disease control, as recurrences may emerge 1–2 years or even longer after surgery; (3) Potential selection bias: the 5 patients were selected from more than 500 laser-treated patients based on strict inclusion and exclusion criteria, and these patients may have more favorable anatomy or better treatment compliance, which may overestimate the efficacy of the combined therapy; (4) Patient satisfaction was not evaluated using validated patient-reported outcome measures (PROMs), which may reduce the objectivity of patient experience assessment.

## Conclusion

This small-sample retrospective exploratory pilot study demonstrates that laser ablation combined with acellular dermal matrix implantation is a feasible and promising minimally invasive strategy for the treatment of recurrent SPD, with favorable short-term outcomes including rapid wound healing, mild postoperative pain, no short-term recurrence and high patient satisfaction in this cohort. These promising results warrant further investigation through long-term, multicenter, randomized controlled trials.

## Data Availability

The raw data supporting the conclusions of this article will be made available by the authors, without undue reservation.
